# Sexual abuse and unwanted pregnancies amongst women and girls in Malawi during the COVID-19 pandemic

**DOI:** 10.1186/s12889-025-25691-9

**Published:** 2025-11-25

**Authors:** Lana Clara Chikhungu, Ugochi Nkwunonwo

**Affiliations:** School of Area Studies, Sociology, History, Politics and Literature, Faculty of Humanities and Social Sciences, University of Portsmouth, Milldam Building, Portsmouth, History PO1 3AS UK

**Keywords:** Sexual abuse, Unwanted pregnancy, Abortion, COVID-19, Defilement, Malawi, Rape

## Abstract

**Background:**

Violence against women and girls increased during the COVID-19 pandemic across the globe. This study investigates the extent to which the COVID-19 pandemic contributed to sexual abuse towards women and girls in Malawi and explores if there was an association between reported cases of sexual abuse (rape and defilement) and the number of women and girls accessing post-abortion care services across Malawi’s four administrative regions: Northern, Central, Eastern, and Southern.

**Methods:**

The study employed an explanatory mixed-methods approach. Administrative data from 2018 to 2021 on reported cases of rape and defilement obtained from the Malawi Police and on the number of women that accessed post-abortion care services from Malawi Public Health facilities was analysed. This was followed by key informant interviews (KIIs) with regional child protection officers and district-level health personnel. Descriptive statistics and bivariate analysis were conducted for the quantitative data, and a thematic analysis was performed for the qualitative data.

**Results:**

During the first wave of the COVID-19 pandemic, school closures were followed by an increased number of reported cases of defilement. Reported cases of rape and defilement increased across all regions except for the Northern region, where a decline was registered. Access to post-abortion care services declined in the Northern and Central regions while slight increases were registered in the Southern and Eastern regions. No association was established between the percentage change in reported cases of defilements and the percentage change in the number of women and girls accessing post-abortion care for the period before and after the COVID-19 pandemic.

**Conclusions:**

Periods of adversity worsen child sexual abuse and lead to a lot of unwanted/unplanned pregnancies and unwanted children. To deal with teenage pregnancies, girls dropping out of school, and poor childbirth outcomes, in addition to programmes that address child marriage and early sexual initiation practices, more interventions should also be directed towards tackling child sexual abuse within the home.

**Supplementary Information:**

The online version contains supplementary material available at 10.1186/s12889-025-25691-9.

## Introduction and background

The movement restrictions to reduce the spread of the coronavirus during the COVID-19 pandemic shed more light on women’s and girls’ vulnerability to violence and sexual abuse within the confines of their own homes [1, 2]. It was, therefore, no surprise that reports in Kenya, India, and Malawi revealed increased levels of sexual abuse, child labour, teenage pregnancies, and child marriages especially amongst children in adversity during the first wave of the COVID-19 pandemic [3–5]. In Malawi, schools were closed for five months from March 2020 as a precautionary measure to prevent the spread of the Coronavirus disease [6]. During this period, Malawi had only recorded very few COVID-19 cases but was under pressure to follow the global COVID-19 prevention protocols of lockdowns and movement restrictions. These school closures, however, inflicted much more harm on the schoolgirls. Apart from the reduced access to education, there were increased levels of teenage pregnancies and child marriages during this period [3]. Between 2019 and 2020, teenage pregnancies increased by 7.8%, and between 2020 and 2021 defilements increased by 22%. In 2021, cases of rape doubled compared to the levels reported in 2018 [7]. These statistics brought to light the potentially high levels of within-home child sexual abuse. To some extent, the increase in teenage pregnancies may also be attributed to the reduced sexual and reproductive health services during the COVID-19 pandemic [8].

Published peer-reviewed work on sexual abuse towards women and girls in Malawi is limited to administrative reports that are not disaggregated by region [7]. However, anecdotal evidence from a Child Protection Officer indicates that reported cases of rape and defilements are higher in the Central and Southern regions compared to the Northern region [9]. Studies that have explored the association between location/geographical factors and sexual violence in Malawi present varied findings. In an analysis based on the 2010 Malawi Demographic and Health Survey, it was established that women of Tumbuka ethnicity (Northern region) were more likely to experience sexual violence than women of Chewa ethnicity (Central region) [10] and that estimates of induced abortion are higher in the Northern region compared the Central region [11]. In contrast, a study based on the 2015 Malawi Demographic and Health Survey data reported a higher likelihood of sexual violence among women from the Central region than among women from the Northern region [12]. The way families in Malawi trace their descendants and the extent to which families value marriage differs across the four administrative regions. Most communities in the Northern region are patrilineal, and upon marriage, the woman relocates to stay with the husband's family. In such communities, children are considered to belong to the father. On the other hand, most communities in the Central, Eastern, and Southern regions are matrilineal and men are expected to live in their wives' villages and build a house there. Most households in the Southern region are female-headed and children belong to the mother [13]. This regional variation in customs warrants a regional-level analysis of all issues of national concern to guide policy and programmatic interventions.

In this study, we employed data on reported cases of rape (sex without consent for women and girls aged above 16 years of age) and defilement (sex with a girl aged less than 16 years with or without their consent) from the Malawi Police and data on the number of women and girls who accessed post-abortion care services between 2018 and 2021 from Malawi Public Health facilities to investigate the extent to which movement restrictions that were implemented to limit the spread of the COVID-19 pandemic may have contributed to the increase of sexual abuse and unwanted pregnancies experienced by women and girls in Malawi*.* The new penal code has recently changed the definition of defilement to a *sexual intercourse with a child, whether or not the child consents.* Where the word “child” means a person under the age of eighteen years [14]. This is a good development because it aligns with the definition of child marriage, and more victims of child sexual abuse will be captured. The term defilement has its roots in the colonial era. Arguably, the term paints a negative picture of the victim, may promote a gender-stereotypical meaning of sexuality, and has the potential to stigmatise the normative development of sexuality in children [15]. Nevertheless, the term is bound to have less psychological impact on victims because it is largely used in official documents. The term that is used publicly is a vernacular translation of the words “*rape of a child*”.

Whilst logically one would expect most unwanted pregnancies to be terminated, the criminalisation of abortion in Malawi entails that most women and girls with unwanted pregnancies end up with unwanted children [16]. A 2015 study that used the Abortion Incidence Complications Methodology established that 53% of pregnancies in Malawi are unintended and that about one-third of them result in termination [11]. This shows that most abortion estimates are likely to be underestimated. Similarly, most of the rape and defilement cases are not reported. It is for this reason that the data on the utilisation of post-abortion care services offer an indirect and credible avenue for tracking sexual abuse in Malawi.

In addition to studying the trend in the reported cases of rape, defilements, and recorded abortions from 2018 and 2021, the study also explores any geographical variations to learn if there is potential for regions to learn from each other. The following research objectives are addressed in this study:

(i) To investigate if there exists a significant monthly variation in number of reported cases of rape and defilement in 2020 and whether the monthly trend aligns with the period of COVID-19 school closures and the reported increases in teenage pregnancies.
(ii) To establish if the number of reported cases of rape and defilement increased across regions in Malawi during the COVID-19 pandemic and provide potential explanations for any regional differences.
(iii) To assess if there exists an association between increases in the number of reported cases of rape and defilement and the number of women and girls accessing post-abortion care services.
(iv) To confirm with regional child protection officers and district health personnel if the patterns and trends obtained from the secondary quantitative data analysis align with their experiences during the study period.

## Methods

### Study design

The study follows an explanatory mixed methods approach [17]. In the first stage, the study employs a retrospective and cross-sectional design by using administrative data from 2018 to 2021 on reported cases of rape and defilement obtained from the Malawi Police and on the number of women who accessed post-abortion care services from Malawi Public health facilities which are available on the DHIS2 website: https://dhis2.org/. DHIS2 is a project developed collaboratively between the HISP Centre at the University of Oslo (UiO) and the global HISP network. The data is provided free of charge as a global public good and is used by more than 80 countries worldwide to analyse health data.

In the second stage, we follow an exploratory qualitative research design by carrying out key informant interviews (KIIs) with seven regional child protection officers from all the seven Malawi Police administrative regions and eight district health personnel from 8 out of the 29 districts ensuring that each region is represented.

### Sample selection

**Fig. 1:**
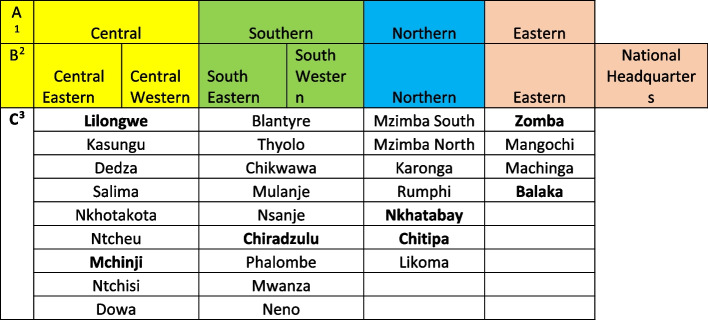
Study areas

As shown in Fig. 1, the four administrative regions comprise of the Northern, Central, Southern, and Eastern regions and the seven Malawi Police Administrative Regions are the National Headquarters, the Northern, Central-Eastern, Central-Western, South Eastern, South Western, and Eastern regional offices, The figure also shows the districts that fall within each administrative region. Please note that there are 29 districts instead of the usual 28 because separate data is provided for Mzimba North and Mzimba South.

Each regional child protection officer or their representative was interviewed as a key informant to verify and seek explanations for the quantitative findings. All seven regional child protection offices were included in the study to ensure national-level representation, making a total of 7 KIIs. We also purposively selected 8 districts for the 8 KIIs from the four administrative regions; Northern (Chitipa and Nkhatabay), Central (Lilongwe and Mchinji), Eastern (Zomba and Balaka), and Southern (Thyolo and Chiradzulu), to provide the district health personnel’s perspective of the quantitative findings derived from the DHIS2 data and to obtain additional complementary contextual information. In total there were 15 KIIs. Purposive sampling was useful to ensure a deliberate selection of participants who were aligned with the aims and objectives of the study [18].

### Procedures

The KIIs with the 7 regional child protection officers were done face-to-face, whilst the KIIs for the 8 district-level health personnel were done via Zoom. One district-level health personnel per district knowledgeable in post-abortion care services was interviewed. The roles of the district health personnel who participated in the study ranged from medical officers, nursing officers, youth-friendly health services coordinators, post-abortion care coordinators, and director of health and social services. Copies of the KIIs guides are provided as supplementary material.

### Inclusion criteria

All regional child protection officers or their representatives and one district health personnel knowledgeable in post-abortion care services from each of the selected 8 districts were included in the study.

### Exclusion criteria

Everyone that was not a regional child protection officer or a district health personnel.

### Sample size for the quantitative data

We extracted district-level data for all districts in Malawi. The total number of cases included in the quantitative data analysis is shown in Table 1. The period 2018 to 2021 nicely captures the period before and after the COVID-19 pandemic, allowing for comparisons between the two periods.

**Table 1:** Total number of reported/recorded cases studied

Year	Rape	Defilement	Post Abortion Care
2018	188	1,539	23,504
2019	214	1,766	21,505
2020	286	2,343	18,723
2021	209	2,497	21,252

### Sample size for the qualitative data

The total sample size for KIIs was 15: 8 district health personnel and 7 regional child protection officers.

### Data analysis

#### Quantitative data

The analysis of quantitative data (reported cases of rape and defilement and the number of women and girls accessing post-abortion care services) involved descriptive statistics, bivariate analysis, and graphical analysis. Rates were obtained per 100,000 women and girls aged 12 or over for each region based on the 2018 population and housing census for the reported cases of rape and defilement and the number of women accessing post-abortion care services from 2018 to 2021.

We calculated the percentage change in reported cases of rape and defilement and the number of women and girls accessing post abortion care services between years using the formula:

$$\left\{\frac{Y2-Y1}{Y1}\right\}\times100.$$

Where Y2 represents the year of interest and Y1 represents the year before.

In 2020, schools in Malawi were closed from March to July to restrict movement and reduce transmissions of COVID-19 infections. Based on reports of increased rape and defilement cases from the Malawi Police and the evidence of an increase in teenage pregnancies from the Malawi Ministry of Gender during the COVID-19 pandemic, we assessed if the monthly reported cases of defilement aligned with the period for school closures due to movement restrictions [6]. This analysis only uses data on defilements since cases of rape are small and further breaking them down for each month does not produce meaningful results. Additionally, defilement cases are more suitable for addressing research objective (i).

We also analysed if the difference in reported cases of defilement between the first half of the year (during the school closure period) and the second half of the year (the period following school closures) was associated with the year of study by undertaking a chi-square Test of Association between the period of the year and the year of study at the national and regional levels.

For the qualitative data (KIIs), the initial step was to transcribe all recordings verbatim. There was no need for translations because interviews were done in English. Once the interviews were transcribed by one of the authors, both authors analysed the data by going through the transcripts and employing inductive coding to identify the key themes and patterns. Thematic analysis is well suited for this study because of its flexibility and ability to provide a rich and detailed complex account of data being analysed [19]. The final step involved determining the common themes emerging from the transcripts and notes taken.

## Findings from administrative quantitative data

### Trends in rape and defilement cases

#### Regional variations

**Fig. 2:**
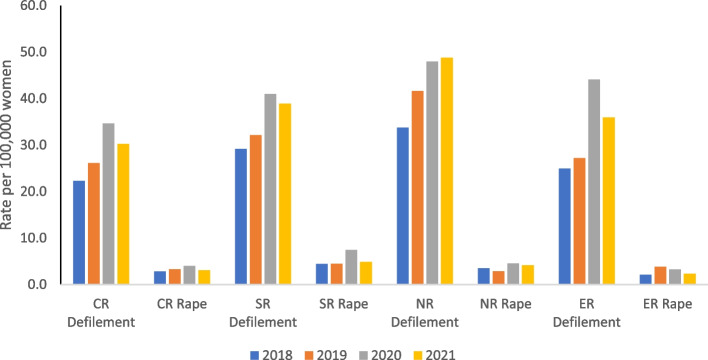
Rate of reported cases of rape and defilement, 2018 to2022 by region per 100,000 women and girls aged 12 +

An analysis of cases of rape and defilement for the years 2018 to 2021 for four regions: Northern (NR), Central (CR), Southern (SR), and Eastern (ER) is presented in Figure 2. There were more reported cases of defilement than cases of rape in all four years. A comparison of cases across the four years reveals that the biggest increase in the reported cases of defilement was between 2019 and 2020. Between 2020 and 2021, the rate of reported cases of defilement decreased slightly in the Central, Southern, and Eastern regions but remained almost the same in the Northern region. The Northern region reported the highest rate of defilement in all four years, and reported cases of defilement were lowest in the Central region.

**Fig. 3:**
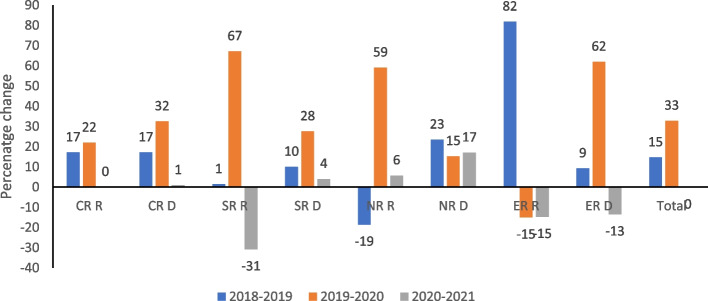
Percentage change in the number of reported cases of rape and defilement by Region, 2018- 2021

Figure 3 shows the yearly percentage change in reported cases of rape and defilement between 2018 and 2021. A percentage change provides a good illustration of change between two periods rather than using actual cases because it compares the amount of change to the level in the previous period. At the national level, reported cases of rape and defilement (combined) increased by 15% between 2018 and 2019 and by 33% between 2019 and 2020. There wasn’t any noticeable change in the cases of defilement and rape between 2020 and 2021. A regional analysis shows that the biggest percentage change in the number of reported cases of rape was in the Eastern region (ER) between 2018 and 2019 (82%), followed by reported cases of rape in the Southern region (SR) between 2019 and 2020 (67%). The biggest increase in defilement cases was registered in the Eastern region in the period 2019 to 2020 (62%) whilst the Northern region (NR) recorded the lowest increase (15%). There was a decline in the number of reported cases of rape across three out of the four regions. In the SR, there was a 31% decline between 2020 and 2021, a 19% decline was registered in the NR between 2018 and 2019, and a 15% decline was recorded in the ER for the periods 2019 to 2020 and 2020 to 2021. The ER was the only region that registered a decline in reported cases of defilement (13%) in the period 2020 to 2021. This is interesting because the ER experienced the biggest increase in reported cases of defilement in the year before (62%).

#### Monthly variations in reported cases of rape and defilement

**Fig. 4:**
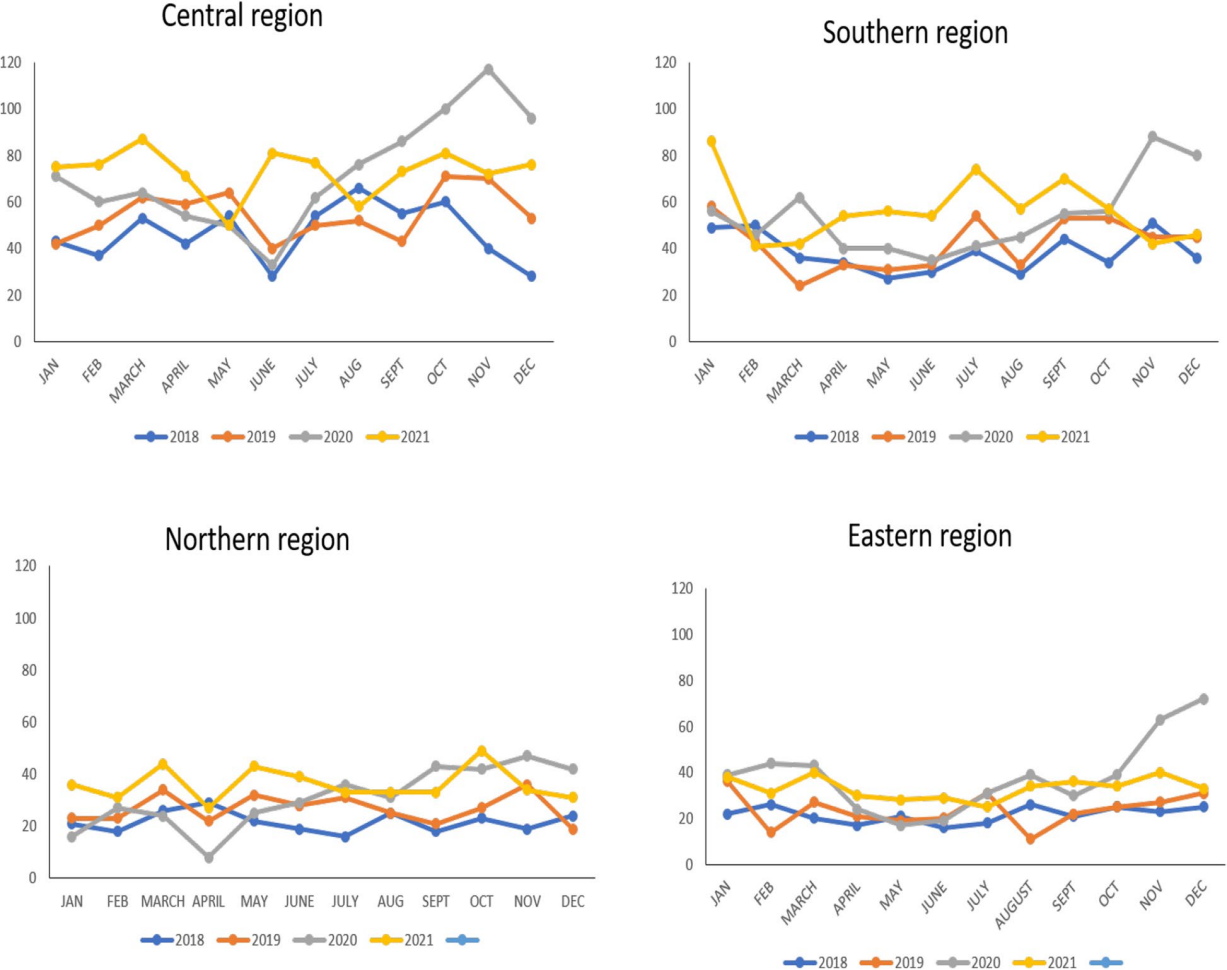
Monthly variations in defilement cases, 2018 to 2021 across the four regions in Malawi

The monthly analysis of reported cases of defilement is presented in Fig. 4 across the four regions: Central, Southern, Northern, and Eastern. The figure shows that the year 2020 stands out of the four years (2018 to 2021) as the year that experienced the highest number of reported cases of defilement in the second half of the year ((July to December) compared to cases reported in the first half of the year (January to June) aligning with reports of increased teenage pregnancies that followed the five months of school closure; March to July.

A chi-square test of Association was undertaken using the data in Table 2 to assess if the difference in the number of cases reported in each half of the year is statistically significant across the four years at the national and regional levels. The results of this statistical analysis show that the *P* value in the test of Association between the Period of Data Collection and the Year of Data collection at the national level, in the Central and Northern regions, was < 0.01; the *P* value for the Eastern region was 0.04, while the P value for the Southern region was 0.07. This indicates that there was a significant association between the number of cases reported in each half of the year and the year the data was collected at the national level, in the Central, Eastern, and Northern regions, but not in the Southern region. All four regions experienced a higher number of reported defilement cases in the second half of the year compared to the first half of the year, but the percentage difference between the year 2020 (COVID-19 pandemic year) and the year before was highest in the Northern region, followed by the Central and Eastern regions. In the Southern region, the percentage for reported cases of defilement in the second half of the year was similar in 2019 and 2020.

**Table 2:** Chi-square test of association between the year and period (Jan to June vs July to Dec) of data collection

Year of Data Collection	January to June	July to December	Total	*P* Value^1^
National Level				
2018	740 (48.08)	799(51.92)	1539 (100)	< 0.01
2019	838 (47.45)	928(52.55)	1766(100)	
2020	926 (39.52)	1417(60.48)	2343 (100)	
2021	1189 (49.81)	1198 (50.19)	2387 (100)	
Total	3693 (45.96)	4342 (54.04)	8035 (100)	
Central				
2018	257 (45.89)	303 (54.11)	560 (100)	< 0.01
2019	317 (48.32)	339 (51.68)	656 (100)	
2020	332 (38.20)	537 (61.80)	869 (100)	
2021	440 (50.17)	437 (49.83)	877 (100)	
Total	1346 (45.44)	1616 (54.56)	2962 (100)	
Southern				
2018	226 (49.24)	233 (50.76)	459 (100)	0.07
2019	222 (43.96)	283 (56.04)	505 (100)	
2020	279 (43.32)	365 (56.68)	644 (100)	
2021	333 (49.04)	346 (50.96)	679 (100)	
Total	1060 (46.35)	1227 (53.65)	2287 (100)	
Northern				
2018	135 (51.92)	125 (49.08)	260 (100)	< 0.01
2019	162 (50.47)	159 (49.53)	321 (100)	
2020	129 (34.86)	241 (65.14)	370 (100)	
2021	220 (50.81)	213 (49.19)	433 (100)	
Total	646 (46.68)	738 (53.32)	1384 (100)	
Eastern				
2018	122 (46.92)	138 (53.08)	260 (100)	0.04
2019	137 (48.24)	147 (51.76)	284 (100)	
2020	186 (40.43)	274 (59.56)	460 (100)	
2021	196 (49.25)	202 (50.75)	398 (100)	
Total	641 (45.73)	761 (54.28)	1402 (100)	

^1^Please note that a *P* value of less than 0.05 indicates that the association between period and year of data collection is statistically significant

### Women accessing post-abortion care before and after the COVID-19 pandemic

**Fig. 5:**
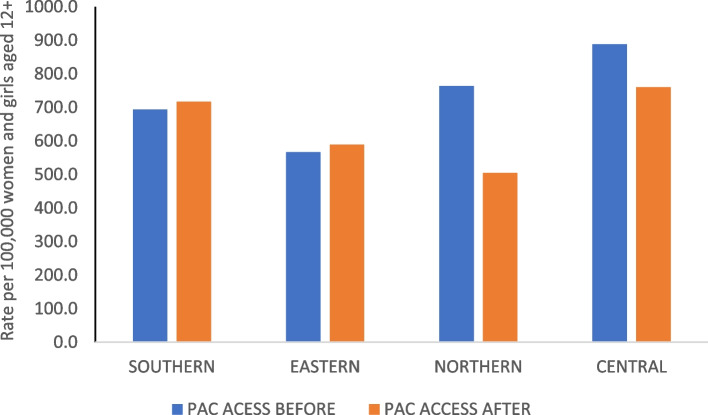
Rate of women and girls accessing post-abortion care services before and after the COVID19 pandemic by region

Figure 5 presents data on women and girls’ access to post-abortion care services by region in the period 2018 to 2021. The figure shows that the Northern and Central regions experienced declines in the rate of women and girls accessing post-abortion care services, with the decline being much higher in the Northern region (−33.9%). The percentage decline for the Central region was −14.3%. The Southern and Eastern regions experienced a small increase in the rate of women who accessed post-abortion care services during the COVID-19 pandemic period; 3.4% and 3.9% respectively. These interesting findings were discussed with district health personnel as part of the KIIs and are reported under Findings from KIIs with district health personnel.

**Fig. 6:**
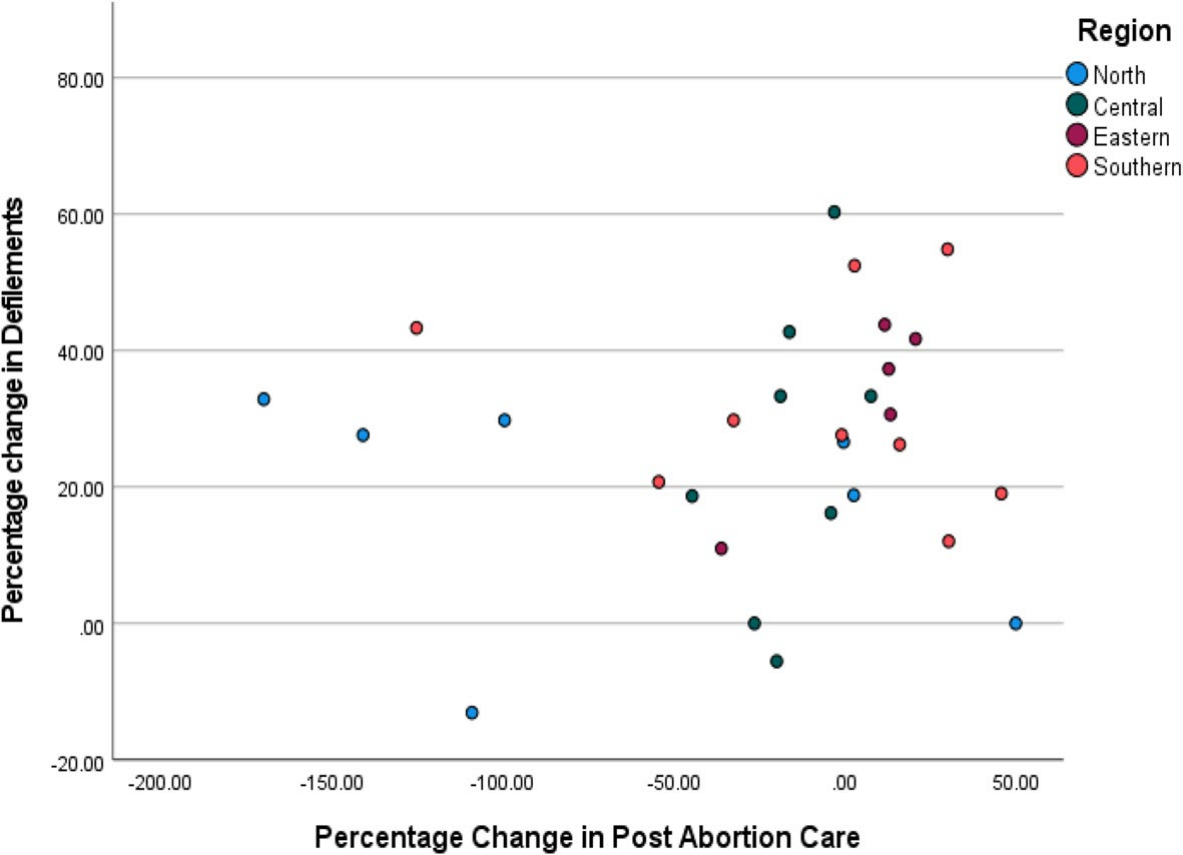
Scatter plot of the percentage change in post abortion care and defilements

To address objective (iii) *assessing if there exists an association between increases in the number of reported cases of rape and defilement and the number of women and girls accessing post-abortion care services*, we carried out a correlation test between the percentage change in the number of women and girls accessing post-abortion care services and defilement. The results showed that the two variables are not correlated: Pearson correlation coefficient 0.137, *P* value (0.496). The association between the two variables is visualised through the scatter plot presented in Fig. 6 using district-level data. The scatter plot does not show any clear pattern of a relationship between the two variables. These findings however should be interpreted with caution due to the limited data points.

## Findings from key informant interviews

### Key informant interviews with regional child protection officers

The key themes that emerged from the interviews with seven regional child protection officers are provided in Table 3. The general view was that the reporting of cases has been increasing in the last four years, possibly due to sensitisation and awareness initiatives by the Malawi Government and non-governmental agencies on sexual violence issues. However, there were mixed views on whether cases increased during the COVID-19 pandemic. Some study participants said that reported cases of rape and defilement had increased during the COVID-19 pandemic period, while others thought it was just a continuation of the increasing trend in the reporting of cases. One regional child protection officer indicated:

*“As I have said that before COVID, the trend has been the same, I am not sure if we could say the rise in cases during this four-year period is attributed to COVID, I would argue that now it has been happening on the same upward scale, but maybe the reporting. The reporting is what has increased so much”. *

It was also reported that most defilements are committed by guardians such as stepfathers, uncles, church elders, or teachers. Peer-to-peer sex (young girls having sex with fellow young boys) is also common but is not reported. Defilements in very young girls aged less than 10 years were said to be associated with ritualistic factors e.g. the belief that such acts can heal men that are HIV positive. For older girls, poverty is a determining factor. One regional Child Protection Officer reported that the trend is that girls report defilement to the police when the man who made them pregnant refuses ownership of the pregnancy. For example, one regional protection officer said:

*“There are several factors to consider, the first one, I would say, is power relations. The perpetrators are people who are supposed to protect the victims. They are more powerful than the victims. For example, an uncle abuses a nephew. The second issue is that to some people, getting married at a young age is normal.” *

### Key informant interviews with the district health personnel

As presented in Table 3, the general perspective was that the number of women and girls accessing post-abortion care services reduced during the COVID-19 pandemic. This aligned with the administrative data presented in Fig. 5. According to some of the interviewees, the reduced access to post-abortion care services was due to the fear of catching the COVID disease. However, some of the interviewees felt that the decline in the number of women accessing post-abortion care services could be explained by under-reporting due to reduced health facility staffing during the COVID-19 pandemic period. One health personnel stated:

*“There are a lot of young women requesting for termination of pregnancy at our facility. We cannot help them so they go to private clinics. I've already admitted that the cases that have been reported are not the true reflection of what is happening because others are still in private clinics, and they are not captured.”*

Through interviews with health personnel from the Southern region, it was established that the likely reason why the Southern region experienced an increase in the number of women accessing post-abortion care services was the increased sensitisation and awareness initiatives taking place in the region. Nevertheless, all district health personnel who participated in the KIIs were of the view that the number of unwanted pregnancies increased during the COVID-19 pandemic. The women and girls who were likely to terminate their pregnancy were: young girls still in school, those with a poor socio-economic status, and married women who were made pregnant by boyfriends while their husbands were away, e.g., whose husbands were away working in South Africa.

**Table 3:** Findings from KIIS with regional child protection officers and health personnel

Key Issues Emerging from KIIS with Regional Child Protection Officers	Key Issues Emerging from KIIS with District Health Personnel
An increasing trend in the reporting of cases	Underreporting of data during the COVID-19 pandemic
Mixed Views on the impact of the COVID-19 pandemic	Reduced access to Post Abortion Care during the COVID-19 pandemic
Most defilements are committed by guardians	Schoolgirls are more likely to terminate pregnancies
Peer-to-peer sex is also common	Increase in unwanted pregnancies
Defilements in very young girls are associated with ritualistic factors	Some married women whose husbands are away may terminate their pregnancies
	Poverty plays a big factor

## Discussion and conclusion

During the first wave of the COVID-19 pandemic, school closures were followed by an increased number of reported cases of defilement. Reported cases of rape and defilement increased during the COVID-19 pandemic across all regions except for the Northern region, where a decline was registered. The increase in cases of defilement in the second half of the year 2020 aligned with reports of an increase in teenage pregnancies by the Ministry of Gender, Children, and Social Welfare and the increase in reported cases of rape and defilement during the COVID-19 pandemic was consistent with the general trend of increased violence towards women and girls during the pandemic across the globe [3–5, 20]. These reports emphasised the increased vulnerability faced by women and girls in times of socio-economic challenges e.g., disasters and pandemics. Most countries in Africa including Malawi did not implement full lockdowns but implemented some movement restrictions such as school closures, closure of international airports, banning public transport which impacted on people’s socio-economic well-being [21].

The Northern region experienced the biggest decline in number of women and girls accessing post abortion possibly due to its unique characteristics. Most communities in the region are far from public services and may have experienced heightened challenges in accessing services during the pandemic, resulting in under-reporting. Under-reporting is also more likely to happen in the Northern region compared to the three other regions because unplanned pregnancies often result in forced marriages [22]. The increase in the number of women accessing post-abortion care services reported in the Southern and Eastern regions was attributed to a high level of sensitisation and awareness initiatives within the area. High levels of sensitisation may also explain the increase in the percentage of reported cases of rape and defilement in 2020 in the Eastern region. Sensitisation and awareness initiatives are important for building confidence in women and girls to access post-abortion care services and report to authorities in cases of defilement or rape [23, 24].

The finding of no association between the percentage change in the number of reported cases of defilements and the percentage change in the number of women and girls accessing post-abortion care for the period before and after the COVID-19 pandemic is interesting but not surprising. This is because abortion is illegal in Malawi. However, there are exceptions when the life and health of a woman or girl are in danger [16]. Despite well-established evidence linking high maternal deaths to unsafe abortion, the Malawi Parliament has missed several opportunities to support amendments to the bill to decriminalise abortion [25, 26].

This is the first study to use administrative Malawi Police data and DHIS2 data to explore the issue of rape, defilement, and unwanted pregnancies at the national level, and it offers a lot of room for improvement. To begin with, the analysis was limited to descriptive, bivariate, and graphical because the data were not disaggregated by important variables such as age, marital status, and socio-economic status to allow advanced statistical analysis such as regression modelling. Despite this limitation, we consider that the explanatory mixed methods design strengthens the validity and reliability of the findings. Caution, however should be observed when interpreting the findings from the KIIs with the district health personnel, as they may not provide a full regional picture. Another limitation is that by only using post abortion care data from Malawi Public Health facilities and not including records of women and girls who were treated in private medical facilities, a huge number of cases from a higher socio-economic class may have been missed [27]. It is estimated that about 40% of health facilities in Malawi are private institutions [28]. The inclusion of data from private medical facilities could help further understand why women of different socio-economic classes terminate their pregnancies, the type of treatment received, and if there are differences in outcomes in these two groups of women. Nevertheless, this study has revealed the patterns and trends for women who access public health facilities. The findings from both the quantitative and qualitative analyses assert previous reports of increased violence towards women and girls during the COVID-19 pandemic [1, 2]. Periods of adversity worsen child sexual abuse and lead to a lot of unwanted/unplanned pregnancies and unwanted children. As part of the implementation the Malawi national strategy to end child marriage [29] policy makers, professional and practitioners should develop or embrace interventions directed towards tackling child sexual abuse within the home.

## Supplementary Information

Supplementary Material 1

## Data Availability

Three sources of data were used in this study, and these can be accessed as follows: 1. Data on the number of women who accessed post-abortion care services from Malawi Public health facilities was obtained from the DHIS2 website: [https://dhis2.org/ ] (https:/dhis2.org ). This is publicly available and can be accessed by writing to the Malawi Ministry of Health. 2. Data on reported cases of rape and defilement can be accessed by writing to the Malawi Police. 3. Data on key informant interviews with regional child protection officers and health personnel can be accessed from the corresponding author on reasonable request and an authorisation from the Malawi ministries of Health and Home Affairs.
