# Supplementary material for: Sexual abuse and unwanted pregnancies amongst women and girls in Malawi during the COVID-19 pandemic
Source: BMC Public Health. 2025 Nov 25;26:14. doi: 10.1186/s12889-025-25691-9 (PMC12764007; doi:10.1186/s12889-025-25691-9)
Supplement: Supplementary file 1 — Supplementary Material 1 [file 12889_2025_25691_MOESM1_ESM.docx]

Guide for Interview with the Officers in Charge of Police Stations

1. Comment on the trend in the number of cases of rape and defilement over the last four years in this district.
2. Do you think that there was an increase in the cases of rape and defilement during the Covid19 pandemic?
3. In your opinion what are the most common circumstances around which rape and defilement occur?
4. What would you say are the key demographic and socio-economic characteristics of the victims and perpetrators of sexual violence?

Guide for Interview with the Directors of Health and Social Services

1. Comment on the trend in the number of women hospitalised due to abortion or termination of pregnancy related issues over the last four years in this district.
2. Do you think that there was an increase in the number of abortion related hospitalisations during the Covid19 pandemic?
3. In your opinion what is the most common reason for women terminating their pregnancy?
4. What would you say are the key demographic and socio-economic characteristics of the women that end up being hospitalised as a result of abortion or pregnancy termination?
